# Psychometric properties of the Exercise Benefits/Barriers Scale in Mexican elderly women

**DOI:** 10.1590/1518-8345.1566.2902

**Published:** 2017-06-05

**Authors:** María Cristina Enríquez-Reyna, Rosa María Cruz-Castruita, Oswaldo Ceballos-Gurrola, Cirilo Humberto García-Cadena, Perla Lizeth Hernández-Cortés, Milton Carlos Guevara-Valtier

**Affiliations:** 1 Doctoral student, Facultad de Organización Deportiva, Universidad Autónoma de Nuevo León, San Nicolás de los Garza, Nuevo León, Mexico. Assistant Professor, Facultad de Organización Deportiva, Universidad Autónoma de Nuevo León, San Nicolás de los Garza, Nuevo León, Mexico.; 2 PhD, Full Professor, Facultad de Organización Deportiva, Universidad Autónoma de Nuevo León, San Nicolás de los Garza, NL, Mexico.; 3 PhD, Full Professor, Facultad de Psicología, Universidad Autónoma de Nuevo León, Monterrey, NL, Mexico.; 4 PhD, Full Professor, Facultad de Enfermería, Universidad Autónoma de Nuevo León, Monterrey, NL, Mexico.

**Keywords:** Psychometrics, Exercise, Nursing, Perception, Validation Studies

## Abstract

**Objective:**

analyze and assess the psychometric properties of the subscales in the Spanish version of the Exercise Benefits/Barriers Scale in an elderly population in the Northeast of Mexico.

**Method:**

methodological study. The sample consisted of 329 elderly associated with one of the five public centers for senior citizens in the metropolitan area of Northeast Mexico. The psychometric properties included the assessment of the Cronbach's alpha coefficient, the Kaiser Meyer Olkin coefficient, the inter-item correlation, exploratory and confirmatory factor analysis.

**Results:**

in the principal components analysis, two components were identified based on the 43 items in the scale. The item-total correlation coefficient of the exercise benefits subscale was good. Nevertheless, the coefficient for the exercise barriers subscale revealed inconsistencies. The reliability and validity were acceptable. The confirmatory factor analysis revealed that the elimination of items improved the goodness of fit of the baseline scale, without affecting its validity or reliability.

**Conclusion:**

the Exercise Benefits/Barriers subscale presented satisfactory psychometric properties for the Mexican context. A 15-item short version is presented with factorial structure, validity and reliability similar to the complete scale.

## Introduction

The scientific literature describes diverging conducts with regard to exercising in the elderly population. Although the majority identifies its benefits, unwillingness to exercise and lack of perseverance continue to exist^1^ ^-^ ^2^. Social determinants have been described that influence the effective practice of this health promotion action^3^, but the understanding of this complex conduct remains insufficient. Particularly in Mexico, the perceptions need to be analyzed that outline the practice of this conduct while aging, as exercising helps to reduce the risk of depression and cognitive deterioration, improves the cardiorespiratory and muscular function and influences the skeletal and functional health of this growing population group^4^.

The generalized prevalence of physical inactivity has led to the need to get to know the reasons or barriers for people to practice this type of conduct or not. People's positive or negative perception of health promotion conducts like physical exercise tends to induce certain behaviors that affect their health. In this respect, the Health Promotion Model explores the factors that influence changes in health behaviors and can be used to analyze the perspectives towards exercising, such as the perceived benefits and barriers^5^. The model explains the relations between individual characteristics and experiences, thoughts and feelings concerning the health conducts and their executions. Two thoughts on the health conducts addressed in the model are: the perceived benefits of the action and the perceived barriers for the action^6^.

The perceived benefits of the action correspond to people's anticipated perception of the positive results of a health conduct. They are based on the personal memories deriving from the background experience or learning by watching others committing themselves to the health action. The individuals invest their time and resources in activities with a high probability of enhancing the experiences with positive results^6^.

What exercising as a health conduct is concerned, the perceived benefits have improved compliance with this conduct as part of the treatment of chronic illnesses^7^ ^-^ ^8^ and have been related with physical-functional improvement, improvement of the neurological condition and decrease of the pain in the elderly population^9^ ^-^ ^11^. In addition, a negative correlation has been found between the perceived benefits and the exercise practice (*r* ^2^=0.16, *p* <.01), suggesting that, although the perceived benefits are clear, the execution of the conduct is limited^12^. Although the adults perceive the importance of exercising in view of their personal background, the belief persists that it could be a waste of time in their daily agenda^13^.

The perceived barriers for the action allude to the negative mental assessments or -imaginary or real- individual impediments that can hinder a commitment to a health conduct. The barriers represents the perceived unwillingness, inconvenience, cost, difficulty or time spent to execute the conduct; they encourage towards the avoidance of the conduct planned. Therefore, when the willness to perform the action is low and the barriers high, executing the conduct will be difficult^6^. The main barriers to exercise identified include the bad climate, the lack of discipline, time, money or company to perform the action^14^. In addition, in adult women of median age, the barriers are health problems, age-related injuries and problems^15^. 

The Exercise Benefits/Barriers Scale [EBBE]^16^ was designed in English to measure these thoughts by Dr. Nola J. Pender in the United States of America. It has been translated and validated in elderly populations in Korea^17^ and Brazil^18^ and, in China, an adapted version was developed and validated to be applied in dialysis patients^19^. The Spanish version was also published by the original authors of the EBBE with acceptable reliability coefficients in Colombia^12^ and Mexico^2^. Nevertheless, no published information has been found on the psychometric properties of the Spanish version. These perceptions might differ in function of the group studied. In addition, variations within the same language might affect the validity of the adapted scales^20^. Therefore, it is relevant to analyze the functioning of the scale in an elderly population in the Mexican context. 

The objective was to analyze and assess the psychometric properties of the subscales in the Spanish version of the Exercise Benefits/Barriers scale in an elderly population in Northeast Mexico. In addition, in a secondary analysis, the feasibility of a short version will be assessed, intended to make it easier to estimate the strength of these perceptions in this population.

## Method

A methodological study was undertaken in a population of 2701 community-based elderly, affiliated with five public centers for senior citizens in the metropolitan area of Northeast Mexico. As the number of men attending these centers is very low, the participants in this study were exclusively women. 

### Participants

Women between 60 and 80 years of age were included, with intact cognitive skills according to Pfeiffer's Questionnaire, able to read and write, without medical contraindication to exercise and who accepted to participate in the study voluntarily. Women who despite the result of the Pfeiffer Questionnaire demonstrated inability to understand instructions were excluded. The sample was calculated using the formula for finite populations and consisted of 329 participants. Simple stratified sampling was used, based on the list of women who attended each of the strata (public centers for senior citizens). 

### Instrument

**Figure 1:**
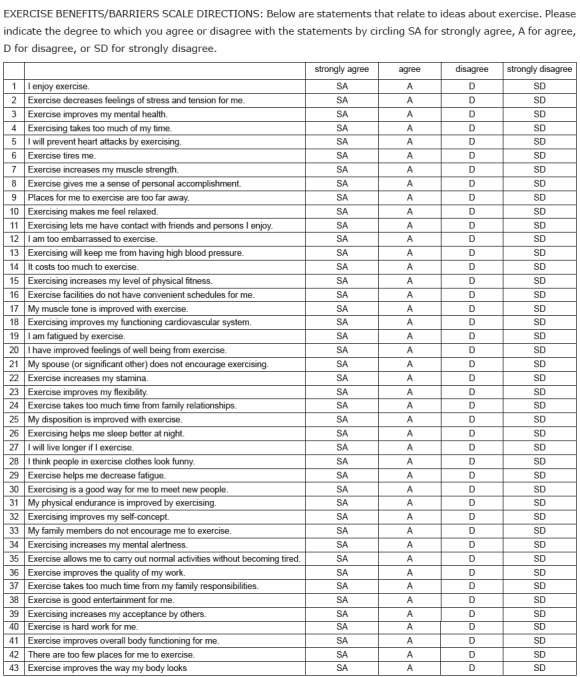
Original version of the Exercise Benefits/Barriers Scale

The Spanish version of the EBBE has been published together with the English version (Figure 1) and was initially translated to Spanish by Juarbe T. It consists of a 43-item quasi Likert scale with four alternative answers. The score "four" corresponds to strong agreement with the assertion, "three" to simple agreement, "two" to simple disagreement and "one" to strong disagreement with the item. The scale includes two subscales: 29 items for the subscale of perceived exercise benefits and 14 for the subscale of perceived exercise barriers. The items of the exercise barriers subscale correspond to assertions 4, 6, 9, 12, 14, 16, 19, 21, 24, 28, 33, 37, 40 and 42. To assess the 14 statements representing the barriers, the answer range varies between 14 and 56; for the benefits, on the other hand, scores range from 29 to 116. In both subscales, a higher score corresponds to a higher perception concerning exercise practice^16^. 

(c) K. Sechrist, S. Walker, N. Pender, 1985. Reproduction without authors' express written consent is not permitted. Permission is obtainable by downloading the Exercise Benefits/Barriers Scale (EBBS) Information and Permission Letter from deepblue.lib.umich.edu. If additional information is needed, contact Dr. Karen Sechrist by e-mail: krsech@pacbell.net.

The scale separately assesses the two perceptions, keeping in mind that, in Pender's model (2011), they constitute two independent and opposed constructs. Sechrist, Walker and Pender (1987) suggest the possibility to assess the complete scale: the result of the exercise barriers subscale is assessed inversely and subtracted from the result of the exercise benefits subscale.

### Procedure

The preliminary analysis included two steps: 1) linguistic and culture review by experts and 2) qualitative pilot study in a small sample of elderly with characteristics similar to those of the final sample. The first step was to review, involving three experts in gerontology nursing, the adaptation of the vocabulary and writing to the Spanish of this Mexican context. The guidelines of the International Test Commission for the adaptation of tests were followed: cultural and language differences, technical aspects and methods and interpretation of results^20^. After collecting the information from the experts, the EBBE was applied to a group of 30 elderly to assess the clarity and adequacy of the measure. As a result of these steps, the formulation of 12 items was modified. 

The study received approval from the institutional ethics committee and from the authorities of the public centers for senior citizens. Properly trained physical exercise professionals collected the data individually and privately while the participants attended the clubhouse. The completion of the EBBE took between five and ten minutes. 

### Data analysis

First, the complete version of the EBBE was analyzed, followed by the separate analysis of the benefits and barriers subscales. The internal consistency was estimated using SPSS version 21.0, by means of Cronbach's alpha coefficient. In addition, to assess the construct validity, the Kaiser Meyer Olkin (KMO) sample adequacy coefficient was used, and the inter-item correlation was assessed in accordance with the theoretical bases of the measuring theory. The factorial structure was estimated by means of exploratory and confirmatory factor analysis in the statistical software AMOS 21.0.

## Results

The participants' average age was 69 years (*SD* =5.44), with 6.5 years (*SD* =2.92) of education. Only 42% confirmed having a partner.

### EBBE - complete version 

The result of the application of Bartlett's test to the correlation matrix among the EBBE items was significant (Bartlett=7168.174, *gl* =903, *p* <.001). The principal components analysis with varimax rotation resulted in two components with an eigenvalue superior to the unit (Determinant=1.120) and rotation in three components. According to the squared sum of the saturations of the rotation, the total value of the first factor was 13.698, representing 31.86% of the variance. The result of the second factor corresponded to 3.542, representing 8.24% of the total variance. The accumulated percentage of the two factors explained 40.09% of the variance. Using .40 as an interpretable saturation criterion in the orthogonal rotation, it is confirmed that the items that saturate in the components correspond to the items proposed in the scale. 

### Psychometric properties per subscale

In the exercise benefits subscale, the KMO adequacy coefficient was significant and acceptable (*KMO* =.959, *p* <.001). According to the squared sum of the saturations of the extraction, the items of this subscale explained 47.23% of the variance. In the exercise barriers subscale, the KMO measure was also acceptable (*KMO* =.751, *p* <.01), with an explained variance coefficient corresponding to 22.97%. 

Internal consistency and item analysis. The alpha coefficient of the exercise benefits subscale corresponded to .958, which is considered very good^21^. A wide range of inter-item correlations was observed, ranging between .235 and .804. The alpha coefficient does not suggest that the elimination of items could enhance the internal consistency of the subscale (Table 1).

**Table 1:** Correlation coefficients and Cronbach's alpha of the exercise benefits subscale in the Exercise Benefits/Barriers Scale. Monterrey, N. L., Mexico, 2015

Item*	Description	Corrected item-total correlation	Cronbach's alpha if the item is eliminated
1	Yo disfruto el hacer ejercicio	.632	.951
2	Hacer ejercicio ayuda a que disminuya mi estrés y tensión	.722	.951
3	Hacer ejercicio ayuda a mejorar mi salud mental	.733	.951
5	Haciendo ejercicio prevengo ataques al corazón	.472	.953
8	Hacer ejercicio me da un sentido de logro personal	.700	.951
7	Hacer ejercicio aumenta la fuerza de mis músculos	.547	.952
10	Hacer ejercicio me hace sentir relajada	.787	.950
11	Hacer ejercicio me permite tener contacto con mis amistades y con personas que me agradan	.576	.952
13	Hacer ejercicio evitará que suba mi presión arterial (hipertensión)	.235	.957
15	Hacer ejercicio mejora mi condición física	.585	.952
17	Mi tono muscular mejora haciendo ejercicio	.668	.951
18	Hacer ejercicio mejora el funcionamiento de mi corazón	.736	.951
20	Cuando hago ejercicio, mi sentido de bienestar mejora	.692	.951
22	Hacer ejercicio aumenta mis energías	.779	.950
23	Hacer ejercicio mejora mi flexibilidad	.804	.950
25	Mi estado de ánimo mejora cuando hago ejercicio	.750	.951
26	Hacer ejercicio me ayuda a dormir mejor por las noches	.623	.952
27	Voy a vivir más tiempo si hago ejercicio	.543	.952
29	Hacer ejercicio me ayuda a disminuir la fatiga	.544	.952
30	Hacer ejercicio es una buena forma para que yo conozca personas nuevas	.690	.951
31	Mi fortaleza física mejora por medio del ejercicio	.727	.951
32	Hacer ejercicio mejora el concepto que tengo de mi misma	.655	.951
34	Hacer ejercicio aumenta mi agilidad mental	.699	.951
35	Hacer ejercicio me permite llevar a cabo actividades normales sin que me canse	.584	.952
36	Hacer ejercicio mejora la calidad de mi trabajo/actividades	.714	.951
38	Hacer ejercicio es buen entretenimiento para mi	.652	.951
39	Hacer ejercicio mejora la imagen general que otros tienen de mi	.627	.952
41	Hacer ejercicio mejora el funcionamiento general de mi cuerpo	.635	.951
43	Hacer ejercicio mejora mi apariencia física	.671	.951

*The item numbers correspond to the numbers attributed in the complete scale.

The alpha coefficient of the exercise barriers subscale was acceptable (.715). Contrary to the item-by-item analysis indicated low correlation coefficients, ranging between .002 and .436. In contrast with the benefits subscale, it was observed that, due to the low corrected item-total correlation (.002), the elimination of item 21 could increase the internal consistency of the barriers subscale to .729 (Table 2). 

**Table 2:** Correlation coefficients and Cronbach's alpha of the exercise barriers subscale in the Exercise Benefits/Barriers Scale. Monterrey, N. L., Mexico, 2015

Item*	Description	Corrected item-total correlation	Cronbach's alpha if the item is eliminated
4	Hacer ejercicio toma mucho de mi tiempo	.348	.679
6	Hacer ejercicio me cansa	.278	.687
9	Los lugares en que yo puedo hacer ejercicio están muy lejos	.393	.672
12	Me da mucha vergüenza hacer ejercicio	.413	.672
14	Hacer ejercicio cuesta mucho dinero	.436	.670
16	Los lugares para hacer ejercicio no tienen horarios convenientes para mi	.418	.668
19	Yo me fatigo cuando hago ejercicio	.311	.683
21	Mi esposo/compañero o ser más querido no me apoya para hacer ejercicio	.002	.729
24	Hacer ejercicio toma mucho tiempo de las relaciones familiares	.375	.677
28	Yo pienso que las personas en ropa deportiva se ven graciosas	.273	.687
33	Mis familiares y amigos no me animan para que haga ejercicio	.235	.695
37	Hacer ejercicio toma mucho tiempo de mis responsabilidades familiares	.388	.674
40	Hacer ejercicio es un trabajo duro para mí	.297	.685
42	Hay muy pocos lugares para que haga ejercicio	.358	.676

*The item numbers correspond to the numbers attributed in the complete scale.

Confirmatory factor analysis. Theoretical and statistical criteria were followed to enhance the internal consistency of the subscales. The distribution of the exercise benefits subscale was not normal (*p* <.05), while that of the exercise barriers subscale was normal (*p* >.05). For the confirmatory factor analysis, the least squares method and the maximum likelihood method were used, in accordance with the distribution of the data.

To assess the goodness of fit of the model per subscale, absolute fit ratios were used (Chi-squared, chi-squared/gl and goodness of fit indices [GFI and AGFI]), incremental fit indices (non-normed fit index [NNFI], parsimony goodness of fit index [PGFI], root mean square error of approximation [RMSEA] or root mean square residual [RMR] when appropriate). A coefficient of 5 or lower is considered to demonstrate good adjustment for the chi^2^/gl index^20^. GFI, AGFI and NNFI coefficients superior to .90 indicate good adjustment^22^. The standardized PGFI coefficients range between 0 and 1. As none of both reaches the limit of .90, coefficients closer to .80 are considered adequate^23^. For the RMSEA, coefficients between .05 and .10 are considered acceptable, and ideal coefficients correspond to 0.08 or less; for the RMR, low coefficients are required, with coefficients closer to zero indicating better adjustment^24^. 

### Secondary analysis to facilitate the application to elderly people

In view of the difficulties to adjust some parameters, the relevance of a factorial solution was analyzed that would be satisfactory for the structural parameters of the model as well as for the validity and internal consistency. In Table 3, the results for the exercise benefits subscale are displayed. The model on the left corresponds to the complete structure of the original subscale, while the model on the right proposes a short version with acceptable goodness of fit parameters. Thus, a six-item version was obtained, with inter-item correlation coefficients ranging between .74 and .82, which could be considered satisfactory. 

**Table 3:** - Factorial analysis of the exercise benefits subscale of the Exercise Benefits/Barriers Scale, 29 and six-item versions. Monterrey, N. L., Mexico, 2015

Benefícios do exercício		29 items	6 items*	Fit
Validity				
	Kaiser Meyer Olkin	.959	.885	>.700
	P-value	<.001	<.001	<.05
Absolute and incremental fit†				
	Chi-squared	362.574	13.859	
	Chi-squared/degrees of freedom	6.251	.990	<5
	P-value	<.001	<.001	<.05
	Goodness of fit index	.989	.997	>.90
	Readjusted goodness of fit index	.987	.993	>.90
	Non-normed fit index	.987	.995	>.90
	Parsimony goodness of fit index	.857	.498	0-1
	Root mean square residual	.022	.011	.05-.10
Reliability				
	Cronbach's alpha	.958	.919	>.70

* Selected items of the exercise benefits subscale: 2, 3, 15, 22, 23, 25; the item numbers correspond to the numbers attributed in the complete scale. †Estimation method: Scale without least squares.

The final version of the exercise barriers subscale consisted of nine items, with inter-item correlation coefficients ranging between .34 and .45. While the model on the left corresponds to the initial structure of the subscale, the model on the right is the solution designed with less items and similar goodness of fit parameters (Table 4). 

**Table 4:** Factorial analysis of the exercise barriers subscale of the Exercise Benefits/Barriers Scale, 14 and 9-item versions. Monterrey, N. L., Mexico, 2015

Exercise barriers		14 items	9 items*	Fit
Validity				
	Kaiser Meyer Olkin	.751	.768	>.700
	P-value	<.001	<.001	<.05
Absolute and incremental fit**^†^**				
	Chi-squared	216.808	64.898	
	Chi-squared/degrees of freedom	2.82	2.40	<5
	P-value	<.001	<.001	<.05
	Goodness of fit index	.916	.960	>.90
	Readjusted goodness of fit index	.886	.933	>.90
	Non-normed fit index	.667	.840	>.90
	Parsimony goodness of fit index	.672	.576	0-1
	Root mean square error of approximation	.074	.065	<.07
Reliability				
	Cronbach's alpha	.715	.722	>.70

Selected items of the perceived exercise barriers subscale: 4,9, 12, 14, 16, 24, 28, 37, 42; the item numbers correspond to the numbers attributed in the complete scale.

†Estimation method: Maximum likelihood.

## Discussion

The reliability results of the Mexican version of the EBBE present essential similarities with the parameters published for the original version^16^. The alpha coefficients of the two subscales of the EBBE presented adequate internal consistency coefficients and were similar to the results obtained in the adaptations in Korea and Brazil^17^ ^-^ ^18^. Considering that the reference point to discuss the results of the adaptation of a scale to a linguistic and cultural context are the related studies^20^; the validity and reliability coefficients found support the use of the EBBE in an elderly population in Northeast Mexico.

The factorial structure and item distribution between the factors of the exercise benefits subscale are in line with the findings for the original version^16^. The high inter-item correlations support the construct validity of this subscale; the discrimination indices can be considered adequate and similar to the findings for the original version.

In contrast, the exercise barriers subscale demonstrated merely acceptable reliability and validity coefficients. This detail was also observed when the original version of the EBBE was applied in an adolescent American population^25^ and in the other adaptations published^17^ ^-^ ^18^. The confirmatory factorial analysis reveals the problem; the low inter-item correlation coefficients suggest the need to review the construct^20^. To give an example, item 21 refers to the "husband or partner's lack of support to exercise", the lack of explanatory power of this item in this sample can be due to the small proportion of participants who signaled having a partner. This explanation could also apply to the case of the adolescent population. As the exercise barriers may depend on aspects directly relate to the population context and culture, the construct needs to be analyzed before making decisions based on this subscale. In short, the EBBE demonstrated a two-factor structure, in accordance with the theoretical principles that guided its construction.

The analysis of the factorial structure of the two subscales revealed that, in this sample, the fit indices AGFI and NNFI of the exercise barriers subscale did not show adequate psychometric properties. This detail suggested the relevance of analyzing the utility of eliminating items to improve these models' goodness of fit parameters. The data are presented as an invitation to reflect on the consideration of this alternative to enhance the estimation fluency of these perceptions in an elderly population.

## Conclusions

The validity and reliability levels found in this sample of Mexican elderly women support the use of the EBBE subscales in the Mexican context. Nevertheless, future studies should analyze the factorial structure of the exercise barriers subscale to corroborate the construct validity before making decisions based on the assessment of this perception. A preliminary analysis revealed that a short version of the EBBE, consisting of only 15 items -six for exercise benefits and nine for exercise barriers- can present a factorial structure, validity and reliability similar to those of the complete scale. The findings for this sample need to be confirmed in elderly populations from other Mexican contexts.
